# Editorial: The society for environmental geochemistry and health (SEGH): 50 years and beyond

**DOI:** 10.1007/s10653-021-01192-7

**Published:** 2022-01-19

**Authors:** M. J. Watts, A. Argyraki, M. Barbieri, A. Brown, M. Button, R. Finkelman, G. Gibson, O. Humphrey, X. Huo, A. S. Hursthouse, B. Kaninga, P. Marinho Reis, D. R. S. Middleton, O. Morton-Bermea, A. Nazarpour, A. S. Olatunji, O. Osano, S. Potgieter-Vermaak, C. Prater, K. Torrance, M. H. Wong, C. Zhang, M. Zia

**Affiliations:** 1 Inorganic Geochemistry, British Geological Survey, Nottingham, UK; 2 Department of Geology and Geoenvironment, National and Kapodistrian University of Athens, Athens, Greece; 3 Sapienza Universita Di Roma, Rome, Italy; 4 SEGH, British Geological Survey, Nottingham, UK; 5 University British Columbia, Kalowna, Canada; 6 University of Texas at Dallas, Richardson, USA; 7 Gibson Consulting and Training, Tarporley, UK; 8 School of Environment, Jinan University, Guangzhou, China; 9 University of the West of Scotland, Paisley, UK; 10 Zambia Agriculture Research Institute, Mount Makulu Central Research Station, P/B 7, Chilanga, Zambia; 11 Departamento de Ciências da Terra, Escola de Ciências, Universidade do Minho, Campus de Gualtarl, Braga, Portugal; 12 Centre for Public Health, School of Medicine, Dentistry and Biomedical Sciences, Queen’s University Belfast, Belfast, UK; 13 Instituto ed Geofísica, Universidad Nacional Autónoma de México, Mexico City, Mexico; 14 Department of Geology, Islamic Azad University, Ahvaz Branch, Ahvaz, Iran; 15 Department of Geology, University of Ibadan, Ibadan, Nigeria; 16 Department of Environmental Biology and Health, School of Environmental Studies, University of Eldoret, Eldoret, Kenya; 17 Manchester Metropolitan University, Manchester, UK; 18 Oklahoma State University, Stillwater, USA; 19 University of Strathclyde, Glasgow, UK; 20 The Education University of Hong Kong, Hong Kong, China; 21 National University of Ireland, Galway, Ireland; 22 Fauji Fertiliser Company Ltd, Rawalpindi, Pakistan

**Keywords:** SEGH, 50th anniversary, Global Diversity

## Abstract

When the SEGH international board released a short editorial paper back in 2019, we described an aim to increase the membership offering, whilst improving the diversity of input regionally, by scientific discipline and to ensure greater and more regular contact across the regions from 2020 onwards. Wider aspirations described in 2019 (Watts et al. 2019) are discussed within this short communication at the end of 2021 to evaluate progress made. In particular, how the SEGH community adapted to the unprecedented circumstances that have challenged each and every one of us throughout the COVID-19 pandemic since early 2020 and are likely to influence our activities for the foreseeable future.

When the SEGH international board released a short editorial paper back in 2019, we described an aim to increase the membership offering, whilst improving the diversity of input regionally, by scientific discipline and to ensure greater and more regular contact across the regions from 2020 onwards. Wider aspirations described in 2019 (Watts et al., 2019) are discussed within this short communication at the end of 2021 to evaluate progress made. In particular, how the SEGH community adapted to the unprecedented circumstances have challenged each and every one of us throughout the COVID-19 pandemic since early 2020 and are likely to influence our activities for the foreseeable future.

**Table:** 

Summary of Future Aspirations described in 2019
Improve engagement with epidemiologists and health practitioners (clinical and Public Health) to improve translation of research into policy
Achieve greater editorial balance of EGAH geographically and to strengthen the ‘Health’ component
Increase presence of epidemiologists and health practitioners (clinical and Public Health) within the conference programmes
Continue to increase regional membership hubs to grow geographical diversity of membership
Grow the Early Career Researcher programme for succession management within SEGH and ‘stay on trend’ with latest research
Evolve relevancy of SEGH goals to include the United Nations Strategic Development Goals to reinforce relevance to policy impact
Increase the membership of business colleagues, in order to encourage research, which is applicable to real-life situations

## Progress to the end of 2021

The COVID-19 pandemic which started in early 2020 and continues to significantly impact life globally at the end of 2021 disrupted plans to increase to two international conferences per year from 2020 in Kenya and China and an increase in smaller regional meetings to improve engagement with the SEGH community. However, as a community, we quickly adapted to online conferences in June 2020 (Watts et al. 2020b) via the first SEGH Live event, which comprised 29 presentations, mainly of five-minute rapid talks with panel discussions and 12-min keynote talks. The short format generated energy, momentum and discussion amongst the 100 or so delegates from 35 countries. The meeting was the first online platform for an SEGH conference and was organised from the UK, scheduled across two days to enable contributions from the Americas and Asia/Pacific time zones to the largely African and European audience. This conference engaged delegates and speakers from countries not normally represented at traditional physical conferences that present logistical financial challenges for participation. The online format also enabled the involvement of a more diverse group, including health professionals that would normally prioritise ‘health’ focussed conferences within their travel plans. The online approach was applied again in November 2020 to deliver online training to the early career researcher (ECR) group, with an SEGH Live2 event repeated in February 2021 to a focused Europe, Africa and Asian audience comprising 20 presentations and an attendance of 70 delegates from 20 countries. Additional regional meetings were organised by the SEGH Americas group in June 2021, to restart the regional section, with 25 delegates for a half-day session of engaging discussion talks from seasoned geochemists and investigation focussed presentations from ECRs. Whilst the pandemic interrupted our aspirations, the switch to online platforms enabled SEGH to adapt and make some progress to meet the 2019 aspirations in a more effective way than if we had relied on in-person meetings via the traditional format. However, we are still challenged in progressing the social interaction which provides the relaxed exchange of ideas and proposals. Sessions for each of the online meetings are recorded and available via www.segh.net. The book of abstracts is available to download for anyone via the Previous Conference Abstract Books page https://segh.net/conference-abstracts.

## Putting the health into SEGH

Table 1 provides some metrics beginning with the 2018 conference in Victoria Falls, Livingstone-Zambia (Watts et al. 2020a) at which time Health was included as a complete session within the physical conferences. As a proportion of presentations, the involvement of health researchers comprised ~ 15–50% between 2018 and 2021. This will provide a baseline point for metrics in future years, although anecdotally, the board agrees that the general input of health professionals has increased since 2018, largely owing to the presentation of projects in the African region encompassing multidisciplinary projects. Such involvement and facilitation by SEGH of this multidisciplinary work will benefit the application and relevancy of environmental geochemistry to global challenges and better demonstrate a pathway to impact, beyond ecological health parameters. This message is reinforced by the ‘One Health’ approach promoted by the World Health Organization, Food and Agriculture Organization of the United Nations and World Organisation for Animal Health advocated multi-sectoral responses to public health threats at the human-animal-ecosystem interface (WHO, 2017). More effort is required to improve the balance within the associate editorial forum for EGAH, with some progress on improving the geographic diversity, with notable additions from Africa so far and the introduction of ECRs to the reviewer pool as an additional third reviewer for future succession training.

**Table 1:** Key performance indicators for SEGH progress on 2019 aspirations. Information from lead authors/topics at conferences would be a simple test of the diversity of information

	2018 Vic Falls, Zambia ORAL (O)/Poster (P)	2019 Manchester, UK ORAL (O)/Poster (P)	2020 SEGH Live ORAL	2021 SEGH Live 2 ORAL	2021 SEGH Live (Americas) ORAL
International collaboration	17 of 44 (O) 8 of 43 (P)	14 of 45 (O) 15 of 56 (P)	12 of 29	10 of 20	1 of 12
ECR presentations versus total in each conference	11 of 44 (O) 12 of 43 (P)	n/a	7 of 29	14 of 20	7 of 12
Public Health content	Complete session devoted to health 6 of 44 (O) 7 of 43 (P)	Complete session devoted to health 16 of 45 (O) 21 of 56 (P)	7 of 29	12 of 20	5 of 12
Non-University organisations, e.g. institutes/business	19 of 44 (O) 25 of 43 (P)	9 of 54	20 of 29	6 of 20	2 of 12
SDG relevance	22 of 44 (O) 15 of 43 (P)	n/a	15 of 29	10 of 20	2 of 12
Pathways to impact	21 of 44 (O) 17 of 44 (P)	n/a	18 of 29	9 of 20	3 of 12
Members in each region, e.g. diversity metrics for delegates	AFRICA 60 AMERICAS 1 ASIA 11 EUROPE 39	AFRICA 12 AMERICAS 4 ASIA 8 EUROPE 83	AFRICA 26 AMERICAS 6 ASIA 7 EUROPE 54	AFRICA 39 AMERICAS 4 ASIA 4 EUROPE 40	AFRICA 3 AMERICAS 18 ASIA 2 EUROPE 6
Emerging issues – is SEGH relevant for the future?	Emerging pollutants, epidemiology in geochemistry and health/cancer and health, agriculture to influence human and animal health/wildlife	New evaluation of health issues. Novel treatments of contaminated land	Emerging pollutants, epidemiology in geochemistry and health/cancer and health, agriculture to influence human and animal health/wildlife	Emerging pollutants, epidemiology in geochemistry and health/cancer and health, agriculture to influence human and animal health/wildlife	Emerging pollutants, exposure and risk, agriculture to influence human and animal health/wildlife, ionomics

N.B. Information used in this table as an example mapped against SDGs 3, 6, 15 and 17, data for conferences evaluated by Watts and Gibson–conference abstracts https://segh.net/conference-abstracts. n/a–not analysed

## Geographical diversity of SEGH

Although the pandemic has had a generally negative effect, in curtailing face-to-face meetings and networking, on a positive note, it has enabled greater geographical diversity. The switch to online delivery of conferences and meetings resulted in more regular engagement with members than the traditional physical annual conference. As already mentioned, the online events attracted delegates and speakers from countries with whom SEGH had no prior engagement, with the online approach providing a more equitable opportunity to participate, particularly for delegates from countries or at an early career stage, for which logistical and financial arrangements are challenging. Africa continued to provide the main point of growth for SEGH, and also a resurgence in the Americas from a very low base beginning to come to fruition attributable to the SEGH Americas meeting in September 2021. In addition, the SEGH Live event inspired a series of conferences co-badged with other societies called ‘The International Student Conferences on Medical Geology and Environmental Health’, starting with a Latin American edition in October 2021. Fifteen students presented to audience of 100 participants covering natural and anthropogenic influences on human, animal and plant health. Future student-oriented conferences will cover sub-Saharan Africa in 2022 and Russia in 2023, providing encouragement and networking opportunities to the students.

After a quiet couple of years from SEGH Asia owing to the absence of physical meetings, an online workshop entitled ‘E-waste Pollution and Public Health’ with presentations given by researchers from across the Asian region in December 2021 restarted engagement with the research community in that region.

Another factor that contributed to the diversity of membership was the introduction of a discounted membership fees for the low- and middle-income countries in 2018. This enabled participation of colleagues and students from these countries to participate directly in SEGH and greatly enriches the international perspective of SEGH. It is hoped that this initiative would be sustained, but also facilitated via funded proposals within the SEGH community to increase engagement and cover the costs for some LMIC participants, as will be discussed in the next section. Overall, the global membership at the end of 2021 has built on the set-up of an African section in 2018 and is now represented across 38 countries compared to 17 in 2019.

## Grow the early career researcher programme

Early Career Researchers were defined as researchers or practitioners within five years of graduation from their last academic degree qualification (e.g. Bachelors, Masters or PhD). The ECR programme has been a growing success, gradually finding its feet with a position paper in Environmental Geochemistry and Health from this cohort to suggest the future priorities for SEGH in 2019 (Humphrey et al., 2021). This group required nurturing and continued engagement to capitalise on early success. Training delivery began to adapt from physical conferences to online delivery, allowing for more regular and diverse inclusion as a positive outcome of the pandemic, with lectures in late 2020 and a writing skills programme funded by the British Academy comprising lectures via online delivery, recorded and available on the SEGH website and group exercises from July 2021 to March 2022. This funding helped to increase the number of countries and ECRs to engage fully with the SEGH community, including access to the official journal, Environmental Geochemistry and Health. The number of ECRs has grown overall to 84 compared to 20 in 2019. The ECR cohort is very much at the heart of SEGH aspirations to improve equitable participation across the regions, stay on trend with the latest research and to provide a succession of leaders to evolve the running of the society.

The ECR cohort had the following aspirations summarised in their joint paper (Humphrey et al. 2020):

- Dedicated ECR sessions at future conferences, organised and chaired by ECRs.
- An ECR special issue in Environmental Geochemistry and Health, with papers contributed and peer-reviewed by ECRs.
- More visibility for winners of the Early Career Researcher Best Paper Award on the journal homepage.
- ECR-led initiatives to give even greater value for money to SEGH memberships.

We have made good progress on the first point, in that a significant proportion of presentations since 2019 was given by ECRs (~ 25–66%). This has been helped by the switch to online delivery, with the sessions in the SEGH Live 2 event chaired by African ECRs. The ECRs have not progressed to a special issue in EGAH, but it is hoped that the ongoing writing workshop for African ECRs will result in a series of papers for submission as a special issue by mid-2022. The ECRs are recognised within each of the presentations, and for the larger meetings with prizes comprising of book tokens provided by SpringerNature up to the value of $250 to first prize and $150 to second prize winners. SpringerNature initiated a process in 2020 to identify papers submitted to EGAH by ECRs and to provide an annual award for the best paper, for which the SEGH international board unanimously voted for Daniel E. González-Santamaría (2019) paper entitled ‘Impact of a tire fire accident on soil pollution and the use of clay minerals as natural geo-indicators’ and was awarded with a $300 book token. To all ECRs or their corresponding authors, when submitting ECR papers, please do tick the box to denote ECR paper. The growth in ECR-led initiatives will need to demonstrate value for money across all regions. The writing workshops have presented ideas for future training initiatives, including writing, communications, networking and a particular demand centred on uncertainty of measurements, critical to demonstrating confidence in any peer review paper, but too often lacking. The ECR group will, in any case, need to take some initiative in developing their own ideas to exploit the opportunity of a global network of ECRs to develop their careers and to speak up to request help and direct training initiatives.

## Relevance of SEGH goals to include United Nations strategic development goals

The simplest metric for this aspiration was to use the number of presentations in recent conferences, including online delivery relevant to the UN SDGs. Table 1 provides a summary of this metric, with projects showing relevance to SDGs in up to half of presentations for any of the conferences, including both oral and poster presentations, demonstrating the relevance for policy impact of the community. Further work could be undertaken to monitor outputs through the SEGH official journal Environmental Geochemistry and Health—an action for the future.

## Increase membership of business colleagues

This has been the most challenging aspiration of all, with the proportion of business colleagues attending or contributing to conference outputs at ~  < 10% of the pool of participants, representing environmental consultancies and traditional sponsor contributions to the science programme, with research institutes, government and non-government agency involvement taking this up to ~ 70% (non-university sector). It may be that the lower number of business colleagues is not a problem and reflects the general involvement in society activities due to differing priorities. However, SEGH should still try to engage with the business community, for similar reasons to targeting the UN SDGs, in maintaining relevance to real-life applications, for example, engagement with professional consultants (air, land, water quality) working with the mining, energy, waste or agricultural sectors.

## Additional outputs for SEGH

The official SEGH journal Environmental Geochemistry and Health (EGAH) has continued to grow in success, with the number of papers submitted increased to 1123 in 2020 and 955 to November 2021 from 610 in 2018, with a 40% acceptance rate, on the back of a rapid increase from 400 + in 2016. The five-year impact factor as one measure of success increased from 3.252 in 2018 to 4.61 for 2020.

As part of the 50th anniversary year of SEGH, a special issue in EGAH is underway comprising of short communication papers for topical discussions, reviews of progress across the multidisciplinary topics within the SEGH community and horizon scanning for future priorities. The papers were drawn from the international board and in particular involving the Fellows of SEGH (FSEGH) established for community members with substantial contributions, who were also connected with ECRs to provide mentorship through involvement in some of the papers. We received nine papers which are in the final stages of review, with a partial release of papers online underway (Davies et al., 2022) with the full issue release delayed into 2022. However, this represents a success both in attracting papers, cross-organisational representation and in connecting ECRs with established researchers in joint activities.

A writing workshop started in July 2021 with 56 trainees from across Africa, with training in earnest since September 2021 through to March 2022 funded by the British Academy. Training delivered by Zoom to trainees from Djibouti, Kenya, Malawi, Niger, Nigeria, Senegal, Zambia in September and November 2021 comprised formal lectures in writing skills from the Editor in Chief of the journals Chemistry Africa and Environmental Geochemistry and Health, along with training regarding uncertainty in measurements. Each of the delegates was led through a process of writing a blog for the SEGH website to practice writing and peer review skills. This cohort will have differing routes to developing their writing skills via short communication or investigative papers in each of the journals, alongside writing skills necessary for communication or in drafting proposals. It is highly likely that this kind of support can continue to be delivered to ECRs on a wider regional basis in the future, using the modified SEGH website platform to host all recorded online presentations and training lectures, free to access, with continued additions to the online resource.

Hybrid meetings are planned for 2022, to celebrate a 50 + 1-year anniversary of SEGH, with an unfortunate delay in a physical celebration akin to the delayed Tokyo Olympics. It is hoped that physical meetings in Kenya and China may be supplemented with international delegates via a hybrid online platform. With the transition to net zero and prospective cuts in carbon emissions, in particular for international travel likely to be imposed, a hybrid format for conferences will be a responsible approach for a society with interests in the protection and investigation of the environment and health. Innovative approaches that continue to evolve the SEGH community interaction may well see groups of delegates within national borders connected virtually to try and boost the essential social interaction that is often missing from the pure online platforms. In any case, the adaptations made during the COVID-19 pandemic will serve SEGH well as the society develops beyond its 50^th^ anniversary year.

We will enter 2022 with a new President and a broader base for the international SEGH board and wider membership. Michael Watts will handover the reins after two 2-year terms following an election of a new candidate by the members. The aspirations presented in 2019 are still relevant for the foreseeable future, but will need to continue to operate within the context of SEGH working to the constraints or exploiting the opportunities arising from the COVID-19 pandemic and presented by global collaboration towards a net zero future for carbon emissions. The current international board was elected through to 2023 providing organisational resilience globally to support the next President, along with formal organisational roles to support the administration of SEGH. The board wish the new President well in guiding SEGH forward to new opportunities.

## References

[CR1] Davies et al. (2022). EGAH editorial- to mark the 50th anniversary of SEGH, Environmental Geochemistry and Health 10.1007/s10653-021-00859-5 10.1007/s10653-021-00859-5 PMC8040013 33844101

[CR2] Gonzalez-Santamarıa DE (2019). Impact of a tire fire accident on soil pollution and the use of clay minerals as natural geo-indicators. Environmental Geochemistry and Health.

[CR3] Humphrey (2021). The society for environmental geochemistry and health (SEGH): Building for the future of early career researchers. Environmental Geochemistry and Health.

[CR4] Watts (2019). The society for environmental geochemistry and health (SEGH): Building for the future. Environmental Geochemistry and Health.

[CR5] Watts (2020). Preface for special issue: Geochemistry for sustainable development. Environmental Geochemistry and Health.

[CR6] Watts, et al. (2020b). SEGH Live and Beyond. *Environmental Geochemistry and Health, 43*, 2799–2801. 10.1007/s10653-020-00722-z 10.1007/s10653-020-00722-z PMC7511523 32970295

[CR7] WHO (2017). One Health, World Health Organisation, https://www.who.int/news-room/questions-and-answers/item/one-health [Accessed online 6th December 2021].

